# Neoadjuvant chemotherapy for borderline resectable and upfront resectable pancreatic cancer increasing overall survival and disease-free survival?

**DOI:** 10.3389/fonc.2022.980659

**Published:** 2022-10-25

**Authors:** Violette Fossaert, Antonio Mimmo, Rami Rhaiem, Linda J. Rached, Mathilde Brasseur, Mathias Brugel, Francesca Pegoraro, Stephane Sanchez, Olivier Bouché, Reza Kianmanesh, Tullio Piardi

**Affiliations:** ^1^ Department of Oncological Digestive Surgery, Hepatobiliary and Pancreatic Surgery Unit, University Reims Champagne-Ardenne, Reims, France; ^2^ Department of Digestive Medical Oncology, University Reims Champagne-Ardenne, Reims, France; ^3^ Division of Hepato-Bilio-Pancreatic, Minimally Invasive, Robotic Surgery and Kidney Transplantation, Department of Clinical Medicine and Surgery, Federico II University Hospital, Naples, Italy; ^4^ Pôle Territorial Santé Publique et Performance des Hôpitaux Champagne Sud, University Reims Champagne-Ardenne, Troyes, France; ^5^ Department of Surgery, Hepato-Bilio-Pancreatic and Metabolic Unit, University Reims Champagne-Ardenne, Troyes, France

**Keywords:** borderline pancreatic cancer, neoadjuvant chemiotherapy, downstaging treatment, pancreatic surgery outcomes, FOLFIRINOX regimen

## Abstract

**Background:**

Pancreatic ductal adenocarcinoma (PDAC) is the most common pancreatic neoplasm. Surgery is the factual curative option, but most patients present with advanced disease. In order to increase resectability, results of neoadjuvant chemotherapy (NAC) on metastatic disease were extrapolated to the neoadjuvant setting by many centers. The aim of our study was to retrospectively evaluate the outcome of patients who underwent upfront surgery (US)-PDAC and borderline (BR)-PDAC, and those resected after NAC to determine prognostic factors that might affect the outcome in these resected patients.

**Methods:**

One hundred fifty-one patients between January 2012 and March 2021 in our department were reviewed. Epidemiological characteristics and pre-operative induction treatment were assessed. Pathological reports were analyzed to evaluate the quality of oncological resection (R0/R1). Post-operative mortality and morbidity and survival data were reviewed.

**Results:**

One hundred thirteen patients were addressed for US, and 38 were considered BR and referred for surgery after induction chemotherapy. The pancreatic resection R0 was 71.5% and R1 28.5%. pT3 rate was significantly higher in the US than BR (58,4% vs 34,2%, p= 0.005). The mean OS and DFS rates were 29.4 months 15.9 months respectively. There was no difference between OS and DFS of US vs BR patients. N0 patients had significantly longer OS and DFS (p=<0.001). R0 patients had significantly longer OS (p=0.03) and longer DFS (P=0.08). In the multivariate analysis, the presence of postoperative pancreatic fistula, R1 resection, N+ and not access to adjuvant chemotherapy were bad prognostic factors of OS.

**Conclusions:**

Our study suggests the benefits of NAC for BR patients in downstaging tumors and rendering them amenable to resection, with same oncological result compared to US.

## Introduction

Pancreatic cancer (PC) is one of the most aggressive solid tumor entities and the fourth leading cause of cancer-related mortality in western countries. It is projected to become the second leading cause of cancer-related death in 2030 (1). Pancreatic ductal adenocarcinoma (PDAC) is the most common histological subtype (>85%) of pancreatic neoplasms. Surgery is the only potential curative treatment of PDAC but, unfortunately, only 20% of patients are eligible for such treatment (2). Indeed, after staging, PDAC is classified into resectable, borderline resectable (BR), locally advanced (LA), or metastatic diseases. Resectable disease is anatomically defined as having the following criteria (i) absence of extra pancreatic disease; (ii) no involvement of the superior mesenteric artery (SMA), hepatic artery, and coeliac axis; and (iii) patency of the superior mesenteric vein (SMV)/portal vein (PV) confluence. Beyond resectable criteria, tumors might remain technically resectable, but surgery carries higher risk of positive margins (R1) with also a higher risk of post-operative complications. Surgery is more challenging and requires frequently associated vascular resection. This might compromise adjuvant treatments and, thus, put patients at a higher risk of recurrence (3). In fact, survival in such patients remains very low, even for those who achieve R0 resection. It is estimated that the 5-year survival rates can hardly reach 20% with more than 80% distant metastatic disease risk (2). Complementary adjuvant treatments are often associated to achieve better OS and DFS (4). Unfortunately, up to 25% of patients with resectable tumors are unable to receive post-operative therapy due to frequent morbidity of pancreaticoduodenectomy and prolonged recovery (5).

These clinical observations suggest that upfront surgery (US) may not be the optimal strategy for BR PDAC. Neoadjuvant chemotherapy (NAC) for BR is becoming the trend in most specialized centers. This strategy has several objectives, (i) the possibility of downstaging the tumoral load to achieve higher rates of R0 resection and (ii) improving the selection of surgical candidates as patients with progressive disease refractory to chemotherapy will not be suitable for pancreatectomy.

Since multiple studies showed encouraging results in metastatic disease with regimens such as FOLFIRINOX (6) and gemcitabine/Nab-paclitaxel (6), many centers extrapolated these results and incorporated these regimens in the pre-operative setting for advanced tumors (7, 8). One study even managed to prove the effectiveness and cost-effectiveness of induction FOLFIRINOX regimen in patients with resectable PDAC (2).

The aim of our study was to retrospectively evaluate the outcome of patients with US-PDAC and BR-PDAC, resected after NAC, and to determine prognostic factors that might affect the outcome in these resected patients.

## Methods

### Study design

**Figure 1:**
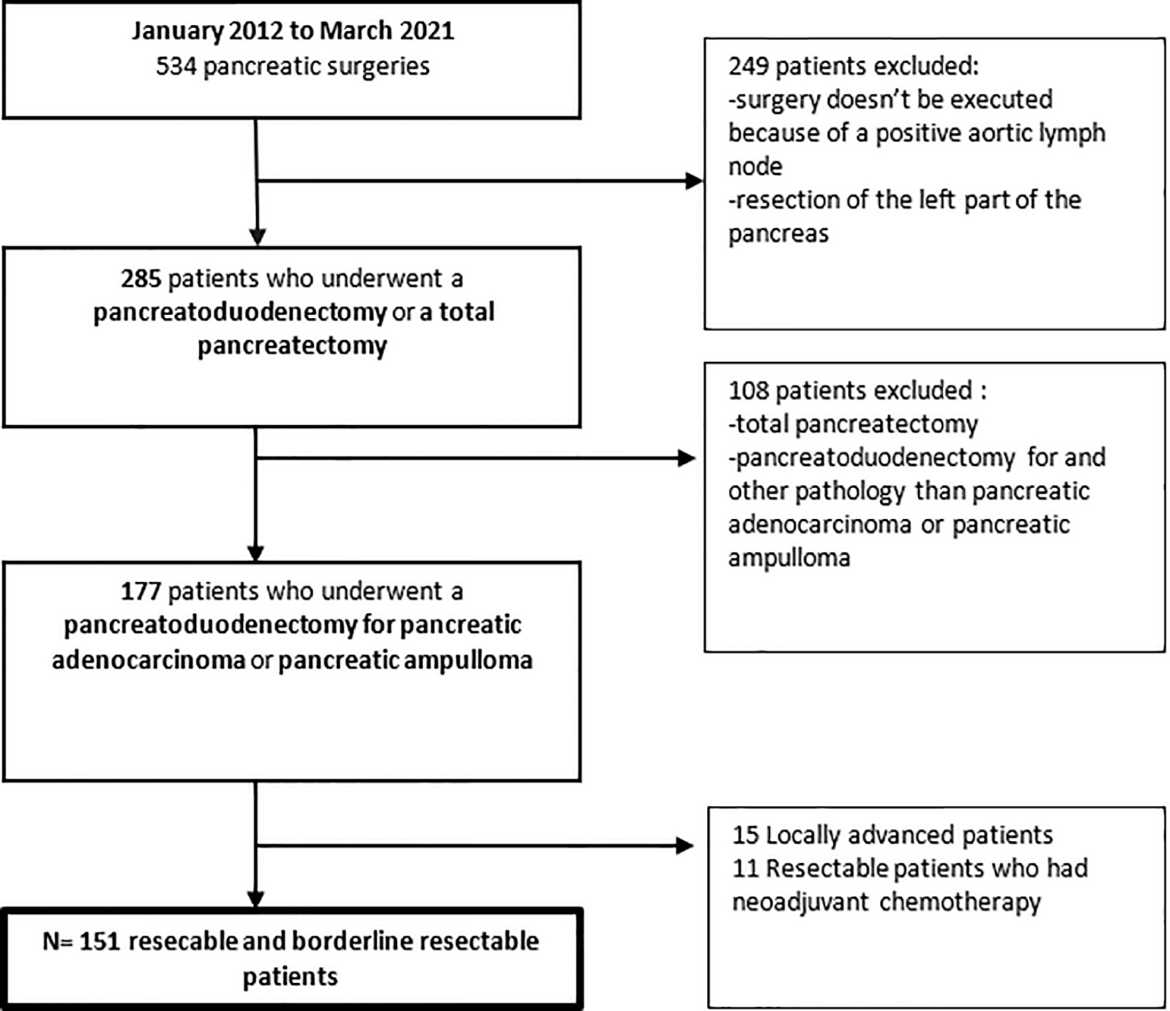
Patients selection and Flowchart.

After the institutional review board approval, all US- and BR-patients at Robert Debre University Hospital between January 2012 and March 2021 were retrospectively identified from institutional databases. Among them, 151 patients were finally selected, 113 US, and 38 patients BR-PDAC who underwent surgery after induction chemotherapy (Flow chart—**Figure 1**).

### Inclusion patients

All patients with PDAC were discussed during our institutional multidisciplinary oncological meeting (MOM). The MD Anderson Cancer Centre (MDACC) classification was used for staging (9). BR patients were referred to pre-operative chemotherapy. Post-chemotherapy reassessment was performed using triple-phase computed tomography (CT) scan and magnetic resonance imaging (MRI) with diffusion-weighted phase. Pancreatectomy was considered in the patients with no newly developed metastases on less than 4 weeks imaging before surgery and who did not experience obvious tumoral locoregional growth.

### Induction chemotherapy

For BR-PDAC, different protocols upon comorbidity were used: (i) modified FOLFIRINOX regimen consisted of oxaliplatin (85 mg per square meter of body-surface area), irinotecan (180 mg per square meter, reduced to 150 mg per square meter after a protocol-specified safety analysis), leucovorin (400 mg per square meter), and fluorouracil (2400 mg per square meter) every 2 weeks; (ii) FOLFOX regimen consisted of oxaliplatin (85 mg per square meter of body-surface area), leucovorin (400 mg per square meter), and fluorouracil (2400 mg per square meter) every 2 weeks; (iii) GEMOX regimen consisted of gemcitabine (850 mg square meter for dose on day 1 and day 8) and oxaliplatin (100 mg square meter for dose on day 2) every 21 days. Patients were monitored for adverse effects and managed mainly in the outpatient clinic.

### Surgery and histopathological evaluation

During laparotomy, once the presence of hepatic metastases and peritoneal carcinomatosis were excluded, harvesting of aorto-caval lymph nodes (LN station 16) was performed. Then, pancreaticoduodenectomy was performed either with the posterior approach or the artery first technique as described by Pessaux et al. (10). Pancreatico-jejunal anastomosis was performed in duct-to-mucosa technique with a stent left in place. Lymphadenectomy was done based on recommendations of the ISGPS (11). All operative sites were drained mostly by unique right-side drains. Pancreatic and bile duct margins were sent for frozen section and re-resection was performed in case invaded margins. On the specimen, the retro-portal lamina was inked to identify the posterior margins. All surgeries were performed by an expert pancreatic surgeon (TP or RK). In our study, we do not separate open pancreatectomy to mini-invasive pancreatectomy, because in the mini-invasive approach, we follow all the steps of open approach, as previously reported (12). Histopathological analysis was performed according to current international TNM classification at the time of resection. We stratified margins into R0 or absence of tumoral contact (margin > 1 mm) and R1 or microscopical tumoral contact (margin ≤ 1 mm) (8). As post-operative complications, we evaluated only the post-operative pancreatic fistula (POPF, grade B or C) (13), delayed gastric emptying (DGE, grade B or C) (14), and post-pancreatectomy hemorrhage (PPH, grade B or C), according to International Study Group of Pancreatic Surgery (ISGPS).

### Follow up

In line with institutional guidelines, all patients are followed after surgery with biological tumoral markers (CA 19.9) and radiological examination (CT scan) every 3 months for the first 2 years and every 6 months thereafter.

### Statistical analysis

For the descriptive analysis, the quantitative variables were expressed as mean and standard deviation; the qualitative variables as numbers and percentages. The Student’s t test was used to compare the quantitative characteristics and the chi-square test for categorical characteristics. The variables were dichotomized, when possible, to facilitate the comparisons. When the Student’s t test could not be used because the variances were not homogeneous, the Mann–Whitney test was applied. When the chi-square test was not valid because the number was lower than 5, the Fisher’s exact test was used. Survival analysis was performed using the Kaplan–Meier and log-rank test method for the endpoints. The variables entered in the Cox model and regression model were those with a univariate p value < 0.20 or clinical significance. The results were expressed as hazard ratio with 95% confidence intervals for the Cox model, and odds ratio with 95% confidence intervals for the logistic regression model. A p value less than 0.05 was considered statistically significant. Statistical analyses were performed using SPSS20.0 (SPSS, Inc., Chicago, IL, USA).

### Ethical considerations

The study design (from retrospective observation) was based on a medical database that did not require patient consent, according to French legislation (15). This study was performed in compliance with national legislation regarding epidemiological studies (Declaration N◦2206749 v 0). Moreover, in accordance with national ethical directives, the requirement for written informed consent was waived because the study was strictly observational and all data were blinded (16). According to the French Public Health Code, this research also did not require an ethical committee. Patients were informed that the study was being carried out *via* the hospital’s registry of ongoing studies.

## Results

Between January 2012 and March 2021, 177 patients underwent duodenopancreatectomy for PDAC. Of those 113 patients who had US for resectable disease, 38 patients had surgery for BR disease after induction chemotherapy (see flowchart—**Figure 1**).

### Patients’ data at diagnosis

Patients’ data at diagnosis are summarized in **Table 1**. Baseline characteristics [age, sex, body mass index (BMI), comorbidity, and American society of anesthesiologists (ASA) score] were statistically similar and they are shown in **Table 1**. Pre-operative biliary drainage and CA 19.9 value were statistically similar and they are shown in **Table 2**. All patients had a PDAC localized in the head of the pancreas or in the uncus or in the peri-ampullary tissue. Mean tumor diameter was 29.3 mm for BR-PDAC patients vs. 23.7 mm for US patients (**Table 2**).

**Table 1:** Baseline population characteristics.

	All patients (n = 151)	Upfront surgery (n = 113)	Borderline (n = 38)	p-value*
**Women**	85	61 (54%)	24 (63.2%)	0.351
**Men**	66	52 (46%)	14 (36.8%)	0.351
**Age at surgery (mean)**	67	67	66	0.607
**Mean BMI (kg/m2)**	24.8	24.9	23.8	0.089
**Diabetes**	39 (25.8%)	33 (29.2%)	6 (15.8%)	0.134
**BMI > 35 kg/m2**	7 (4.6%)	6 (5.3%)	1 (2.6%)	0.680
**High blood pressure**	64 (42.4%)	49 (43.4%)	15 (39.5%)	0.708
**Weaned or active smoking**	57 (37.7%)	42 (37.2%)	15 (39.5%)	0.431
**ASA I**	19 (12.6%)	16 (14.2%)	3 (7.9%)	0.4
**ASA II**	70 (46.4%)	51 (45.1%)	19 (50%)	1
**ASA III**	46 (30.5%)	32 (28.3%)	14 (36.8%)	0.538
**ASA IV**	1 (0.7%)	1 (0.9%)	0	1

*Comparison between the Upfront Surgery and the Borderline.

**Table 2:** Neoadjuvant data.

	All patients (n = 151)		Upfront surgery (n = 113)	Borderline (n = 38)	p-value*
**Tumor size (mean) (mm)**		25.2	23.7	29.3	0
**Biliary drainage**		97 (64.2%)	71 (62.8%)	26 (68.4%)	0.564
**Endoscopic drainage**		89 (58.9%)	67 (59.3%)	22 (57.9%)	1
**Biliary prosthesis**		85 (56.3 %)	61 (54%)	24 (63.2%)	0.351
**CA19.9 <37 U/ml**		32 (21.2%)	24 (21.2%)	3 (21.1%)	0.308
**CA19-9 (median and extremes)**		103 (0.8-19648)	103 (0.8-19648)	83 (2.1-12000)	
**Neoadjuvant chemotherapy**		38 (25.2%)	0	38 (100%)	0
FOLFIRINOX**		35 (23.2%)	0	35 (92.1%)	
**GEMOX**		3 (2%)	0	3 (7.9%)	

*Comparison between the Upfront Surgery and the Borderline group.

**Three patients changed for FOLFOX because of a bad tolerance.

### Chemotherapy data

BR-PDAC patients’ data following chemotherapy are shown in **Table 2**. Induction chemotherapy alone with no radiotherapy. All BR-PDAC patients received pre-operative chemotherapy, 92.1% (n=35) FOLFIRINOX regimen, and 7.9% (n=3) GEMOX regimen (**Table 2**). In addition, 3 patients (8.5%) experienced severe side effects of irinotecan with FOLFIRINOX and were switched to FOLFOX. Moreover, 32 patients (91.5%) who completed neoadjuvant therapy tolerated their treatment without hospital admission or emergency department care. The median duration of neoadjuvant treatment was 2.1 months, with an average of 5.2 cures per patient. The mean reduction in tumor size was 28.7%, from a median size of 30 mm to 20 mm (**Table 3**). Surgical exploration was performed 5–6 weeks following chemotherapy completion. The 78.8% of patients (119) received an adjuvant chemotherapy regimen. **Table 5** summarizes the data of the different protocols used. Often, a different protocol was used due to toxicity problems or a compromised performance status. The median duration of adjuvant chemotherapy was 5.1 months, with an average of 6.8 cures per patient.

**Table 3:** After neoadjuvant chemotherapy data.

Borderline n = 38	Before neoadjuvant chemotherapy	After neoadjuvant chemotherapy
**Tumor size (mean) (mm)**	29.2***	19.9****
**Tumor size (median and extremes) (mm)**	30 (15-50)	21 (0-40)
**Regression (mean) (mm)**		8.5
**Regression (median and extremes) (mm)**		6 (0-25)
**Percentage of regression (mean)**		29.9%
**CA19.9 < 37 U/ml**	7 (18,4%) *	11 (28.9%) **
**CA19.9 (mean)**	1072.2*	241.7**
**CA19-9 (median and extremes)**	83 (2.1-12000) *	36 (2.1-2101.6)

*5 missing data, ** 5 missing data, *** 2 missing data, **** 3 missing data.

### Post-operative data

The rate of venous resections was significant higher in the BR patients than in the US patients (52.6% vs. 23%, p=0.001) (**Table 4**).

**Table 4:** Pathological data on post-resection specimens.

	All resected patients (n = 151)	Upfront surgery resected (n = 113)	Borderline resected after NAC (n = 38)	p-value*
**Venous resection**	46 (30.5%)	26 (23%)	20 (52.6%)	0.001
**Tumor size (mean) (mm)**	26.3	27.2	23.5	0.143
**R0 >1 mm**	108 (71.5%)	86 (76.1%)	22 (57.9%)	0.039
**R1**	43 (28.5%)	27 (23.9%)	16 (42.1%)	0.039
**T0**	1 (0.7%)	0	1 (2.6%)	0.441
**T1**	19 (12.6%)	11 (9.7%)	8 (21.1%)	0.089
**T2**	40 (26.5%)	25 (22.1%)	15 (39.5%)	0.054
**T3**	79 (52.3%)	66 (58.4%)	13 (34.2%)	0.005
**T4**	11 (7.3%)	9 (8%)	2 (5.3%)	0.731
**N+**	104 (68.9%)	80 (70.8%)	24 (63.2%)	0.687
**Number of N+ (mean)**	2.9	3	2.6	0.552
**Collected lymph nodes (mean)**	19.6	20.4	17.5	0.104
**LN ratio (mean)**	0.14	0.15	0.13	0.552
**Venous emboli**	79 (52.3%)	61 (54%)	18 (47.4%)	0.574
**Perineural sheathing**	108 (75.5%)	82 (72.6%)	26 (68.4%)	0.679
**Lymph emboli**	85 (56.3%)	67 (59.3%)	18 (47.4%)	0.257

*Comparison between the Upfront Surgery and the Borderline group.

Histopathological and post-operative data are summarized in **Table 4**. Resection quality rate showed 71.5% R0 and 28.5% R1. Concerning tumor size, T3 rate was significantly higher in the US than BR (58.4% vs. 34.2%, p= 0.005). Lymphadenectomy resulted in mean of 19.6 lymph node per procedure with a positive rate of 70.8% and an average of two involved LN per patient in the BR-PDAC, vs. 63.2% and one involved LN in US patients. Comparisons between POPF, PPH, and DGE rates were not significative in two groups (**Table 5**). As expected, POPF rate was 11.5% in the US patients vs. 5.3% in the chemo-inducted patients. Moreover, 15 patients had POPF during the post-operative period. All these patients received post-operative Somatostatin analogues for at least 7 days. Among the four patients with a grade C pancreatic fistula, two died during the first 90 post-operative days due to PPH, and two needed a redo-surgery during the same hospitalization. One of the patients with a grade B pancreatic fistula died during the first 30 post-operative days due to a mesenteric ischemia.

**Table 5:** Post-operative data.

	All patients (n = 151)	Upfront surgery (n = 113)	Borderline (n = 38)	p-value*
**Grade B-C pancreatic fistula**	15 (9.9%)	13 (11.5%)	2 (5.3%)	0.131
**Grade B-C gastroparesis**	19 (12.6%)	12 (10.6%)	7 (18.4%)	0.258
**Grade B-C hemorrhage**	14 (9.3%)	10 (8.8%)	4 (10.5%)	0.752
**D30 mortality**	6 (4%)	4 (3.5%)	2 (5.3%)	0.642
**D90 mortality**	11 (7.3%)	7 (6.2%)	4 (10.5%)	0.272
**1-year survival**	113 (88.4%)	89 (78.8%)	24 (63.2%)	
**3-year survival**	38 (25.1%)	37 (32.7%)	8 (21.1%)	
**Mean survival (month)**	29.4	30,3	26.6	
**Mean survival (day)**	894	921	809	
**6 months tumor recurrence**	10 (6.6%)	7 (6.2%)	3 (8.3%)	
**1-year recurrence**	44 (29.1%)	35 (31%)	9 (236%)	
**3-year recurrence**	77 (60%)	57 (50.4%)	20 (52.6%)	0.851
**Mean DFS (day)**	495	471.5	456	
**Mean DFS (month)**	15.9	15.5	13.5	
**Adjuvant chemotherapy**	119 (78.8%)	88 (77.9%)	31 (81.6%)	0.819
**FOLFIRINOX**	32 (26.9%)	16 (18,1%)	16 (51,6%)	<0.001
**GEMZAR + XELODA**	74 (62,1%)	68 (77,2%)	6 (19,4%)	<0.001
**LV5 FU2**	6 (5%)	1 (1,1%)	5 (16,1%)	<0.001
FOLFOX **, ***	7 (5,9%)	3 (3,4%)	4 (12,9%)	0.053

*Comparison between the Upfront Surgery and the Borderline group.

**Patients who had neoadjuvant FOLFOX went on an adjuvant therapy with FOLFOX.

***Bad tolerance for FOLFIRINOX because of post-operative complication or bad general condition.

Among the patients, 19 had DGE during the post-operative period. A medical treatment was managed for all these patients in first place, with the administration of prokinetic drugs sometimes associated with a nasogastric tube. A nasojejunal tubes were necessary for three patients. One of them was reoperated at the 21^st^ post-operative day due to an early stenosis of gastrojejunal anastomosis.

In addition, 14 patients had PPH. Two patients had a parietal bleeding that was controlled by surgical hemostasis. Two patients had a bleeding from a hemorrhagic ulcer of the gastrojejunal anastomosis. One of them received a surgical hemostasis, and in the other the bleeding was spontaneously interrupted. A portal vein bleeding was the cause of the death for two patients, despite redo-surgery for hemostasis. Two patients had a bleeding from the superior mesenteric artery, treated by radiological embolization followed by surgical hemostasis, in one of them. Bleeding came from fissure of proper hepatic artery pseudoaneurysm in two patients, and radiological embolization and stenting were performed in both patients, the post-procedure outcome for one of them was fatal. Three patients with sentinel bleeding, without cause, detected to arteriography. The last patient had a bleeding from a branch of superior mesenteric artery treated by radiological embolization. In the cohort patients, six patients died in the first 30 post-operative days and a total 11 patients died during the first 90 post-operative days. Mortality was for the first 30 post-operative days, four (3.5%) in the US patients vs. two (5.3%) in the BR groups (p=0.64). For the first 90 post-operative days, seven (6,2%) in the US patients *vs*. four (10.5%) in the BR groups (p=0.27). Among the six patients who died within the first 30 post-operative days, one died due to a pulmonary embolism, two patients died because of a hemorrhagic shock, one of multiple organ failure after a PPH, one had a several cardiac arrest, and one died of a mesenteric ischemia. Among the five other patients who died within the first 90 post-operative days, two died because of a mesenteric ischemia, two died because of multiple organ failure after a PPH. For one of these patients, the reason of the death is unknown.

### Survival and recurrence

**Figure 2:**
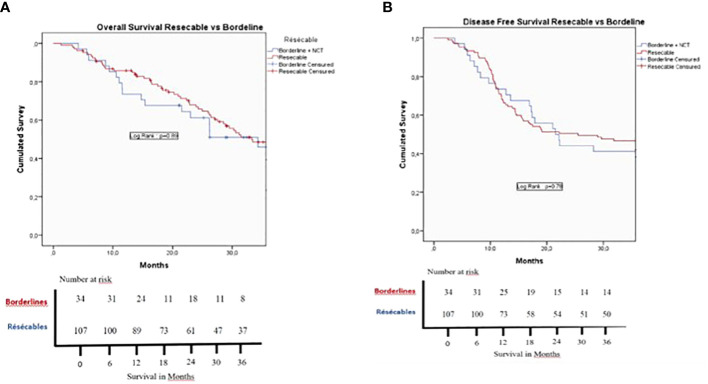
Kaplan–Meier survival curves for survival rates. **(A)** Overall survival and **(B)**. Disease- free survival for the Upfront surgery group (red curve) and the Borderline group (blue curve). There is no significant difference for OS (p=0.,89) and DFS (p=0.,78) between the two groups. Patients diedead at the 90th post-operative day were excluded of the survival analysis.

**Figure 3:**
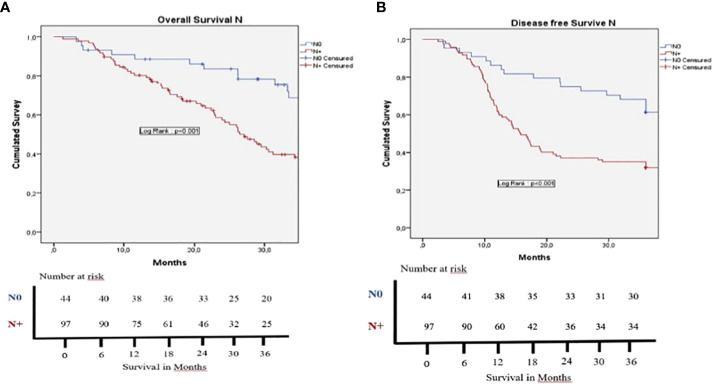
Kaplan-Meier survival curves for survival rates. **(A)** Overall survival and **(B)** Disease free survival for the N+ (positive collected lymphe nodes at the pathology analysis) group (red curve) and the N0 (no positive collected lymphe node at the pathology analysis) group (blue curve). There is a significant difference for OS (p < 0,001) and DFS (p < 0,001) between the two groups. Patients dead at the 90th post-operative day were excluded of the survival analysis.

**Figure 4:**
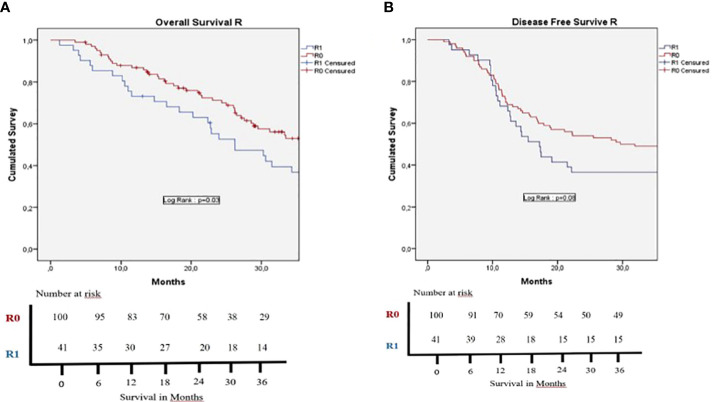
Kaplan-Meier survival curves for survival rates. **(A)** Overall survival and **(B)** Disease free survival for the R0 (resection margin > 1mm at the pathology analysis) group (red curve) and the R1 (resection margin < 1mm at the pathology analysis) group (blue curve). There is a significant difference for OS (p =0,03) and no significant difference for DFS (p =0,08) between the two groups. Patients dead at the 90th post-operative day were excluded of the survival analysis.

In the cohort population, 78.8% of patients received adjuvant CHT, 77.9% in the US vs. 81.6% in the BR patients (p=0.82). As shown in **Table 5**, the global mean post-operative OS was 29.4 months, whereas median post-operative DFS was 15.9 months. **Figure 2** shows no statistically significant difference in OS between US and BR patients. BR patients vs. US patients 1 and 3 years OS were not statistically significant, 73.5% and 23% vs. 85.8% and 23.6%, respectively. This was also not significant for 1 and 3 years DFS, 73.5% and 38.2% vs. 68.2% and 42.1% for BR patients vs. US patients (p=0.89), respectively. No statistically significant difference OS and DFS was evidenced in the US group based on the value of Ca19.9 considering a cutoff of 120 U/ml (p=0.76 in OS and P=0.26 in DFS) or 500 U/ml (p=0.62 in OS and p=0.96 in DFS). **Figure 3** shows the results according to the nodal invasion (N0 vs N+). A statistically significant difference in OS was observed after 1 and 3 years in OS for N0 versus N+ patients, 88.5% and 58.4% vs. 80.3% and 32.4% (p=0<001). For the 1 and 3 years DFS, a statistically significant difference was observed, 86.4% and 61.4% vs. 61.9% and 32% between N0 vs. N+ patients (p<0.001). In **Figure 4**, the outcome according to the status of margin invasion (R0 vs. R1) is shown. A statistically significant difference in OS at 1 and 3 years was observed between R0 and R1 patients, 86.8% and 46.4% vs 73.2% and 29% (p=0.03). However, no statistically significant difference was observed in DFS at 1 and 3 years between R0 and R1 patients, 70% and 46% vs 68.3% and 29.3% (p=0.08). When we stratify all the variable that can influenced the OS and DFS in the cohort population, we found that in the multivariate analysis that POPF, R1, N+ and not access to adjuvant chemotherapy were bad prognostic factors of OS (**Table 6**); and we found that PPH, N+ were bad prognostic factors of DFS (**Table 7**).

**Table 6:** Univariate and multivariate Cox-regression analysis of the overall survival with Borderline group and Upfront surgerygroup.

Variable	Cohort	Univariate *p* value	Multivariate HR	95% CI	*P *value
**Women**	85	0.562			
**Biliary drainage**	97				
**Endoscopic drainage**	89				
**Biliary prosthesis**	85				
**ASA1**	19				
**ASA 2**	70				
**ASA3**	46				
**High Blood Pressure**	64				
**Diabetes**	39				
**Weaned or active smoking**	51				
**BMI >35kg/m2**	7				
**Ca 19.9 < 37 U/ml**	32				
**Neoadjuvant chemotherapy**	35				
**B-C pancreatic fistula**	25	0.048	3.746	1.073-13.086	0.038
**B-C hemorrhage**	14	0.269	3.170	0.318-31.565	0.325
**B-C Gastroparesis**	19				
**R1**	43	0.036	2.716	1.268-5.818	0.010
**Positive collected lymph nodes**	104	0.036	2.695	1.068-6.797	0.036
**Venous emboli**	79	0.238			
**Perineural sheathing**	108	0.466			
**Lymph emboli**	85	0.300	0.712	0.363-1.4	0.325
**T1**	19	0.005			
**T2**	40	0.016			
**T3**	79	0.011			
**T4**	11	0.114			
**Venous resection**	46	0.121	0.478	0.201-1.136	0.095
**Adjuvant chemotherapy**	119	0.045	3.485	1.226-0.904	0.019

**Table 7:** Univariate and multivariate Cox-regression analysis of the recurrence with Borderline group and Upfront surgery group.

Variable	Cohort	Univariate *p* value	Multivariate HR	95% CI	*P *value
**Men**	66	0.508			
**Women**	85	0.508			
**Biliary drainage**	97	0.393			
**Endoscopic drainage**	89	0.317			
**Biliary prosthesis**	85	0.139			
**ASA1**	19	0.323			
**ASA 2**	70	0.299			
**ASA3**	46	0.071			
**High Blood Pressure**	64	1			
**Diabetes**	39	0.455			
**Weaned or active smoking**	51	0.862			
**BMI >35 kg/m2**	7	0.699			
**Ca 19.9 < 37 U/ml**	32				
**Neoadjuvant chemotherapy**	35	0.846			
**B-C pancreatic fistula**	25	0.787			
**B-C hemorrhage**	14	0.009	3.29	1.014-10.67	0.047
**B-C Gastroparesis**	19	0.329			
**R1**	43	0.106			
**Positive collected lymph nodes**	104	0.002	0.395	0.223-0.7	0.001
**Venous emboli**	79	0.194			
**Perineural sheathing**	108	0.003	0.613	0.346-1.086	0.094
**Lymph emboli**	85	0.250			
**T1**	19	0.006	1.604	0.593-4.341	0.352
**T2**	40	0,353			
**T3**	79	0.002	0.783	0.482-1.273	0.324
**T4**	11	0.532			
**Venous resection**	46	0.477			
**Adjuvant chemotherapy**	119	0.001	1.226	0.612-2.458	0.565

## Discussion

Our study showed that mean OS and DFS in BR patients after NAC and in the US patients were 26.6 and 13.5 months vs. 30.3 and 15.5 months, respectively. In the BR patients, the tumor diameter dropped after pre-operative chemotherapy significantly, with a mean percentage of regression of 29.9%. No evidence of tumor was seen on the control CT scan for five patients. In the two groups, the rate of post-operative pT3 tumors was significantly higher in the US patients (p= 0.005), while after NAC, BR patients, who were initially in more advanced tumor status, had a similar OS and DFS of US patients at 1–3 years (p=0.89 and p=0.78). In our cohort, according to nodal status, the patients had a significant better OS and DFS when they did not have a nodal infiltration by the tumor (N+) (p<0.001). Equally, according to margin status, the patients had a significant better OS when there was a microscopic tumoral invasion of the margin (p =0.03) and a non-significant better DFS when there was a microscopic tumoral invasion of the margin (p =0.08).

Most of the patients with PDAC present with locally advanced or metastatic disease, in fact only 15%–20% present with upfront resectable disease. To date, the only potentially curative therapy for PDAC remains surgical resection. NAC is increasingly used to target occult disease if present, select patients, and possibly downstage tumors.

Induction chemotherapy for borderline tumors is acquired but it is place for resectable borderline. PDAC is not standardized and its role is not well definite for the different results reported by the literature (17–19).

Most of the patients in our study were highly selected, most had good performance status (OMS ≤ 2), with no contraindications to NAC, especially vascular anatomical abnormalities for subsequent major pancreatic surgery, and BR patients received pre-operative induction chemotherapy mainly FOLFIRINOX regimen (84.2% of patients). Despite its high toxicity profile often necessitating dose re-adjustments or change of regimen in frail patients; FOLFIRINOX has proven superiority over other regimens in many studies, mainly the ACCORD trial that showed prolonged survival with minimal impairment in quality of life in well-selected patients (6). In the borderline group, only the 52.6% of patients received vascular resection. This point was marked during the latest international consensus, given that the major determinants of resectability in PDAC remain anatomical findings on imaging (mainly size and vessel involvement), biologic behavior of the tumor (Ca 19-9), and the patient’s characteristics (OMS and co-morbidities) (20). By carefully selecting patients, our study showed how NAC succeeded in downstaging tumors and affecting biological behavior, while preserving a good performance status allowing patients to undergo a highly morbid surgical procedure like a pancreaticoduodenectomy.

In order to study the effect of NAC on survival, follow up was continued post-operatively, from the histopathologic study of surgical specimens to the surgical morbidity and mortality. Patients were then followed with markers and imaging every 3 months for 2 years post-operatively and every 6 months thereafter.

In our series, even if in the borderline group, the tendency is to have fewer pancreatic fistulas; there is no significant difference compared to the US (5.3% vs. 11.5%). This is mainly due to a lack of statistical power, but the tendency is clearly towards fewer POPF after neoadjuvant treatment. In the literature, the results are conflicting. Cools et al.’s data using the ACS-NSQIP–targeted pancreatectomy from 2014–2015 showed a statistically significant difference in terms of Type C pancreatic fistula between patients that received NAT and US patients (21). Denbo et al. considering all types of pancreatectomy (Whipple et. DP), no difference was found between patients that received NAT and US patients (P = 0.96) (22). Extremely interesting are the results of the study by Marchegiani et al. that reports the experience of the Verona team. In fact, NAT significantly reduces the incidence of pancreatic fistula (P = 0.05), but based on the Modified Accordion Severity Grading System and average complication burden (ACB) used to compare the patients treated with NAT with the patients who underwent US, the results show that the patients who develop a fistula post-NAT are associated with an increase in clinical burden (23–27). These results introduce, in our opinion, an aspect that is often overlooked, the toxicity of chemotherapy. The toxicity of FOLFIRINOX grade 3/4/5 can reach up to 50% (ASCO 2022). This often results in surgical management of fragile patients who may have a more complicated post-operative course. Although NAT allows us to operate on patients with a “hard” pancreas and better selected (exclude patients who develop metastases during chemotherapy), on the other hand, the pre-operative management requires multidisciplinary management.

This aspect of patient fragility also results in difficult access to adjuvant chemotherapy. As we have well shown in our results, among the OS risk factors, pancreatic fistula and lack of access to adjuvant chemotherapy are themselves negative risk factors.

Since multiple series showed that radical surgical resection with negative margins is the key to achieve better survival, margins were noted in all specimens, especially the retroperitoneal margin. A minimum of 1-mm margin has been adopted by the current Royal College of Pathologists’ guidelines for pancreatoduodenectomy specimens (28). In fact, many studies showed that the survival benefit of negative margin was lost when the tumor was within 1 mm of the resection margin (R1 < 1 mm). Our study showed an R0 resection in 76.1% of US patients vs. 63.2% of BR patients, R1 (margin inferior to 1 mm) in 11.5% of US patients vs. 15.7% of BR patients and R1 (microscopical contact with the tumor, margin 0 mm) in 12.4% of US vs. 21.1% of BR patients. In pancreatic surgery, R0 resection is generally reported to be achieved in 70%–80% of cases, but, unfortunately, the definition of R0 resection is not yet worldwide standardized. When 1-mm margin was used, R0 resection rate dropped to 5%–26% (29–33). A meta-analysis of 19 studies by Chandrasegaram et al. found that the rate of R0 resection with a 0-mm margin was 72%, while that with a 1-mm margin was 41% (34). Yamamoto et al. noted a drop in R0 resections after the revised classification from 84% to 43% (35). Chang et al. reported on 365 patients, 46% of whom were resected with a margin wider than 1.5 mm. Patients with a margin wider than 1.5 mm were actual long-term survivors, as compared to a margin of less than 1.5 mm (36). In our series, in the 76.1% and 63.1% of resected patients (US and BR patients), the resection margin was ≥ 1 mm. One of the reasons that can explain high rate of R0 resections was likely achieved due to the artery first approach, common in our technique. As described by Pessaux et al., resection starts by isolating the mesenteric artery at the origin and along its upper/right border in contact with the adventitia allowing us to dissect the artery up to the last fat cell and thus gaining margins (10).

The aim of our study was to assess the effect of NAC on BR pancreatic tumors compared to the patients that received US and how these changes might affect OS and DFS. As expected, a significant effect on tumor size were observed in histopathological post-operative analysis: higher T3 rate was found in the US patients, despite a mean lower size of the tumor shown on pre-operative CT scan. Our study did not show any significant difference, concerning OS and DFS between two groups. In our series, patients with R1 resection had worse OS than patients with R0 resection (p=0.03). At the same time, N+ patients had a worse OS and DFS at 1–3 years when compared to N0 patients (p<0.001 respectively), the points that are largely admitted in the literature and recently by Netherlands studied showing the effect of margin and lymph node status in all pancreatectomies for cancer (37).

In our study, we analyze prognostic factor that can have an impact on OS such as presence of POPF, R1 margin, presence of nodal tumoral invasion (N+), and absence of adjuvant chemotherapy as being bad prognostic factors at multivariate analysis. Bilici et al. showed that median survival time was better in R0-resected patients when compared with R1-resected patients (22 months *vs*. 15 months) (38). Li et al. analyzed retrospectively prognostic factor that impacted OS and showed that R1 and N+ were important prognostic factors for OS after pancreatic resection. Moreover, the authors found a statistical difference in OS for the patients that have POPF (p<0.05) (39). In the study of Girgis et al., multivariate analysis predicting overall survival, the absence of adjuvant chemotherapy negatively impacted the OS (P < 0.001) (40). Recently, Strobel et al. reviewed all patients undergoing upfront resection for resectable and borderline-resectable PDAC between 2001 and 2011. The extent of lymph node involvement was the strongest predictor of 5 years OS. Patients with pN0R0 had a 5-year OS rate of 38.2% (41); in our experience, patient with pN0R0 had a 3-year OS rate of 70%.

Our study had several limits. The retrospective design from one center, and limited number of included patients especially for BR-disease. No intention-to-treat analysis was performed. The previous results might suggest the benefit from such strategy in highly selected patients. We also admit the presence of few missing data that we were not able to retrieve and that may alter the interpretation of the result.

In conclusion, the present study confirms the favorable outcomes of radical pancreatectomy for BR-patients after NAC. This seems to allow significant downstaging of BR-patients both in tumors size and LN with similar 1 and 3 years OS and DFS when compared to US patients. In the lack of prospective randomized trials, our policy is to propose US for resectable and routine NAC for BR tumors. The artery-first technique seems to help achieving better R0 margin rates.

## Data availability statement

The raw data supporting the conclusions of this article will be made available by the authors, without undue reservation.

## Author contributions

VF and AM are 2 first co-authors because they have contributed in same manner to the development of work. (I). Conception and design: TP, AM, RR, VF, RK (II). Administrative support: AM, VF, LR, MBa (III). Provision of study materials or patients: TP, OB, RK (IV). Collection and assembly of data: AM, RR, VF, LR, MBa (V). Data analysis and interpretation: AM, RR, TP, SS, OB, RK (VI). Manuscript writing: All authors (VII). All authors contributed to the article and approved the submitted version.

## Conflict of interest

The authors declare that the research was conducted in the absence of any commercial or financial relationships that could be construed as a potential conflict of interest.

## Publisher’s note

All claims expressed in this article are solely those of the authors and do not necessarily represent those of their affiliated organizations, or those of the publisher, the editors and the reviewers. Any product that may be evaluated in this article, or claim that may be made by its manufacturer, is not guaranteed or endorsed by the publisher.
